# 

**DOI:** 10.1192/bjb.2022.79

**Published:** 2023-10

**Authors:** Katherine Elizabeth Witter

**Affiliations:** Logandene dementia assessment and treatment unit, Hertfordshire Partnership University NHS Foundation Trust, Hemel Hempstead, UK. Email: k.witter@nhs.net

**Figure:**
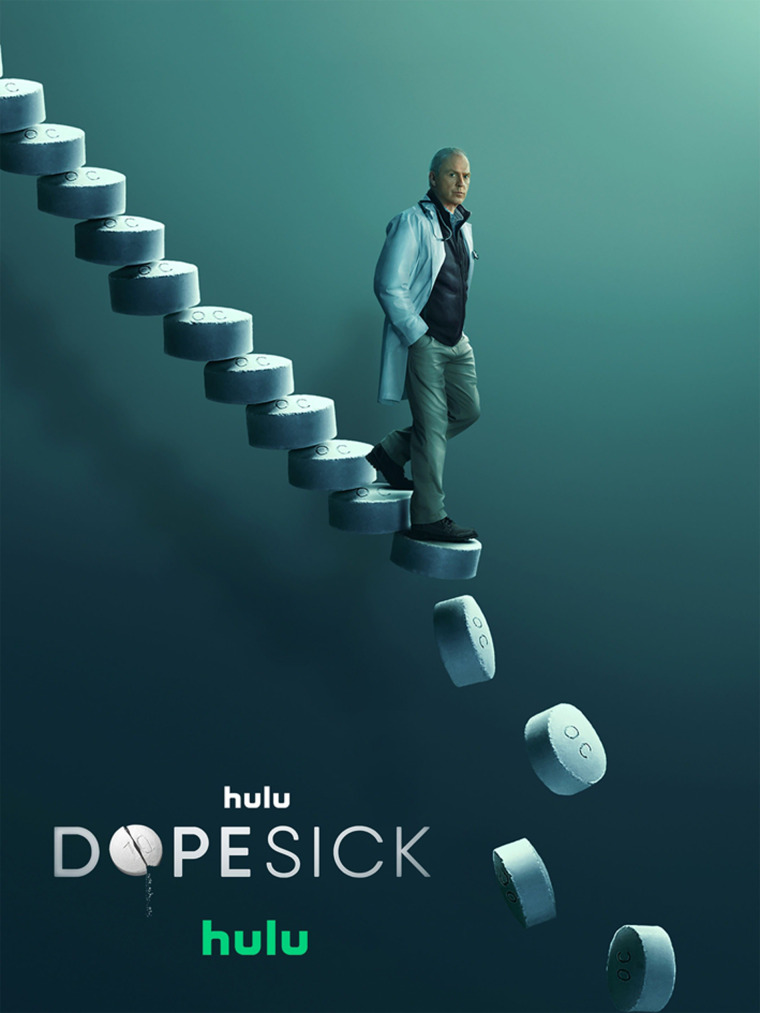

*Dopesick* is an eight-part drama series that premiered on Hulu in 2021 and is currently available on Disney+. It is an examination of the opioid crisis unleashed and driven in the USA in a large part by the painkiller OxyContin (oxycodone), relentlessly promoted by Purdue Pharma, an American privately held pharmaceutical company.

Owned by the Sackler Family, Purdue Pharma started promoting OxyContin in the mid-1990s, claiming the product to be ‘slow release’, ‘non-addictive’ and with low misuse potential. It became the most popular analgesic in the USA. The company began by targeting small towns where chronic pain injuries were common, such as industrial mining and logging areas. Purdue Pharma persistently endorsed, mis-marketed and sold their opioid to physicians, who they misinformed with falsely represented data and aggressive sales techniques. After successfully encouraging widespread prescriptions and use of their drug, it is now widely believed to have caused a public health crisis through misuse and dependence. Its use has led to extensive crime, desperate addicts and devastated communities. Purdue Pharma was dissolved in 2021 after pleading guilty to criminal charges related to its marketing of OxyContin.

Telling a factually correct story but using fictionalised details, *Dopesick* illustrates the ordeal of opioid-related disorders from different patients’ perspectives, particularly dependence and the classic cluster of behavioural, cognitive and physiological phenomena that develop with repeated substance use. The relatable characters include a female miner, Betsy, who initially requires analgesia for painful physical injury and faces significant personal and family difficulties. She is treated by a well-intentioned but lonely family doctor, Dr Samuel Finnix. Dr Finnix is initially reluctant to prescribe OxyContin because of the well-documented addictive qualities of opioids. He is, however, persuaded by an eager young Purdue Pharma rep named Billy to try a ‘new’ drug for his struggling patients. Billy convinces the physician that this medication has minimal misuse potential and excellent, long-lasting effects. After widely prescribing to those in his care, Dr Finnix starts misusing the substance himself, exploits his position as a clinician and spirals into a desperate situation of dependence.

Along with other characters, Betsy and Dr Finnix clearly demonstrate desire to take the drug, inability to control its use, persistence in use despite harmful consequences, prioritising taking the drug over other parts of their lives, tolerance and clear withdrawal state. This includes being ‘dopesick’, a slang term for symptoms experienced in withdrawal. The individuals demonstrate the struggle to recover from addiction, the challenges and impact of relapse, the desperation and the wider devastation to families and communities that can ensue.

The vivid visual depictions give the audience, myself included, an appreciation of the strength it takes to recover from opioid-related disorders, along with the sad and easy ability to become caught up in them in the first place. Although I cannot personally comment on how realistic the portrayals are, from experience and research the series seems an honest and relatable representation of opioid dependence and promotes reflection on the role of large pharmaceutical companies, which remain a hugely profitable industry in the USA.

